# Supplementary material for: Paramutation at the maize pl1 locus is associated with RdDM activity at distal tandem repeats
Source: PLoS Genet. 2024 May 30;20(5):e1011296. doi: 10.1371/journal.pgen.1011296 (PMC11166354; doi:10.1371/journal.pgen.1011296)
Supplement: S1 Fig — (A) Structure of a single copy of the Pl1-Rhoades penta-repeat. Cyan box: unique subregion (USR) used as a hybridization probe. Arrows represent DNA transposons (light gray), Helitrons (black), and LTR retrotransposons (dark gray). BglII (B) and NsiI (N) restriction sites producing hybridizing fragments in the Pl1-Rhoades (B) and pl1-B73 haplotypes (C) are shown. Black boxes represent gene models, large arrows represent the penta-repeat sequences, cyan boxes represent the USR, and green shading represents the region of recombination for pl1-R30. (D) Southern blot of Pl1-Rhoades (Pl1-Rh), pl1-B73, and pl1-R30 genomic DNA digested with BglII and NsiI and probed with a radiolabeled USR fragment. (PDF) [file pgen.1011296.s001.pdf]

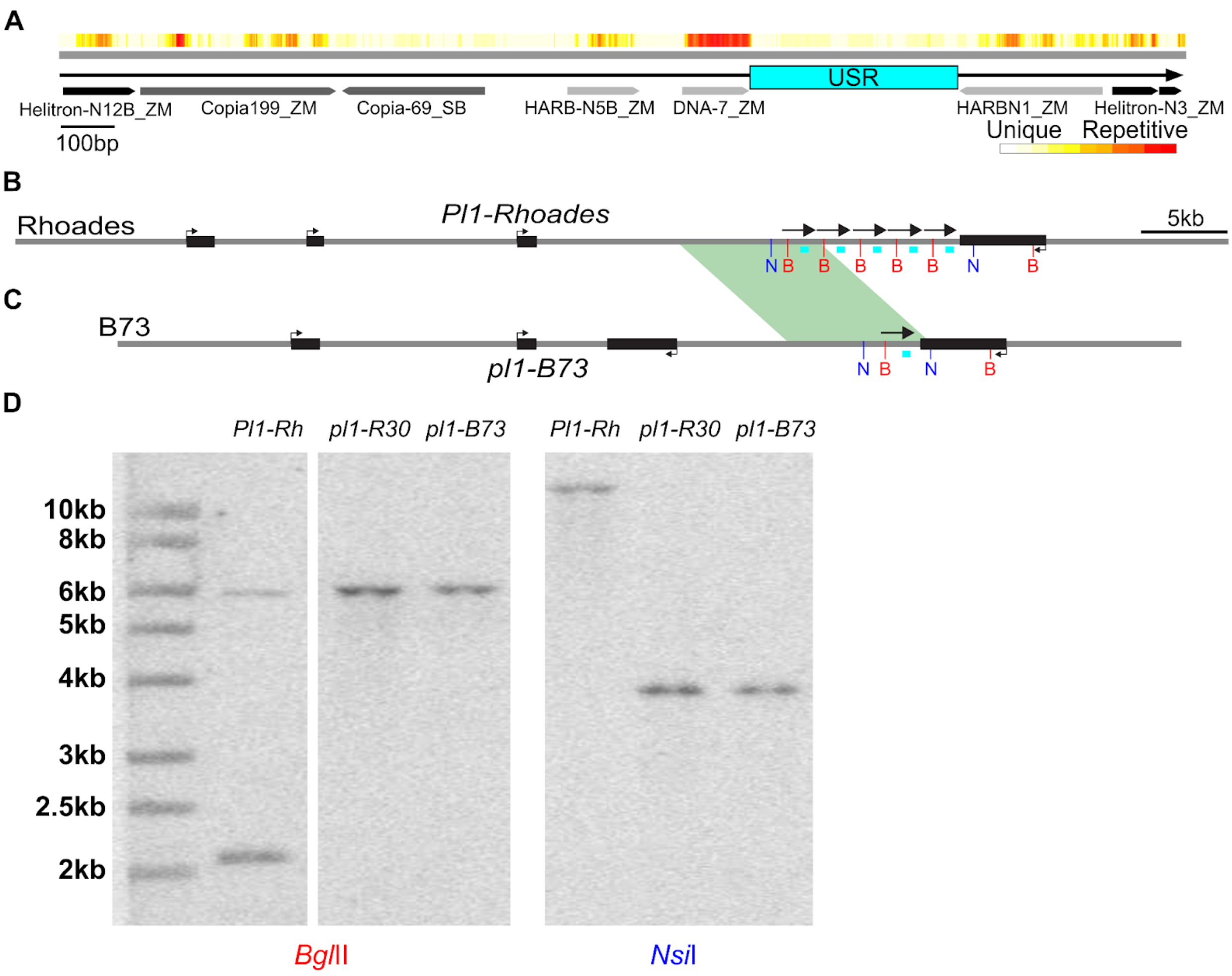

S1 Fig. Structures of *pl1* haplotypes

(A) Structure of a single copy of the *Pl1-Rhoades* penta-repeat. Cyan box: unique subregion (USR) used as a hybridization probe. Arrows represent DNA transposons (light gray), *Helitrons* (black), and LTR retrotransposons (dark gray). *Bgl*II (B) and *Nsi*I (N) restriction sites producing hybridizing fragments in the *Pl1-Rhoades* **(B)** and *pl1-B73* haplotypes **(C)** are shown. Black boxes represent gene models, large arrows represent the penta-repeat sequences, cyan boxes represent the USR, and green shading represents the region of recombination for *pl1-R30*. **(D)** Southern blot of *Pl1-Rhoades (Pl1-Rh)*, *pl1-B73*, and *pl1-R30* genomic DNA digested with *Bgl*II and *Nsi*I and probed with a radiolabeled USR fragment.
