# Supplementary material for: Paramutation at the maize pl1 locus is associated with RdDM activity at distal tandem repeats
Source: PLoS Genet. 2024 May 30;20(5):e1011296. doi: 10.1371/journal.pgen.1011296 (PMC11166354; doi:10.1371/journal.pgen.1011296)
Supplement: S2 Fig — Mean fold pl1 mRNA (2-ΔΔCt) in Pl-Rh and Pl' inner seedling and husk leaves relative to inner husk leaves from Pl' individuals measured by qRT-PCR normalized to actin1 levels in biological duplicate plants from A619 and B73 lines. (PDF) [file pgen.1011296.s002.pdf]

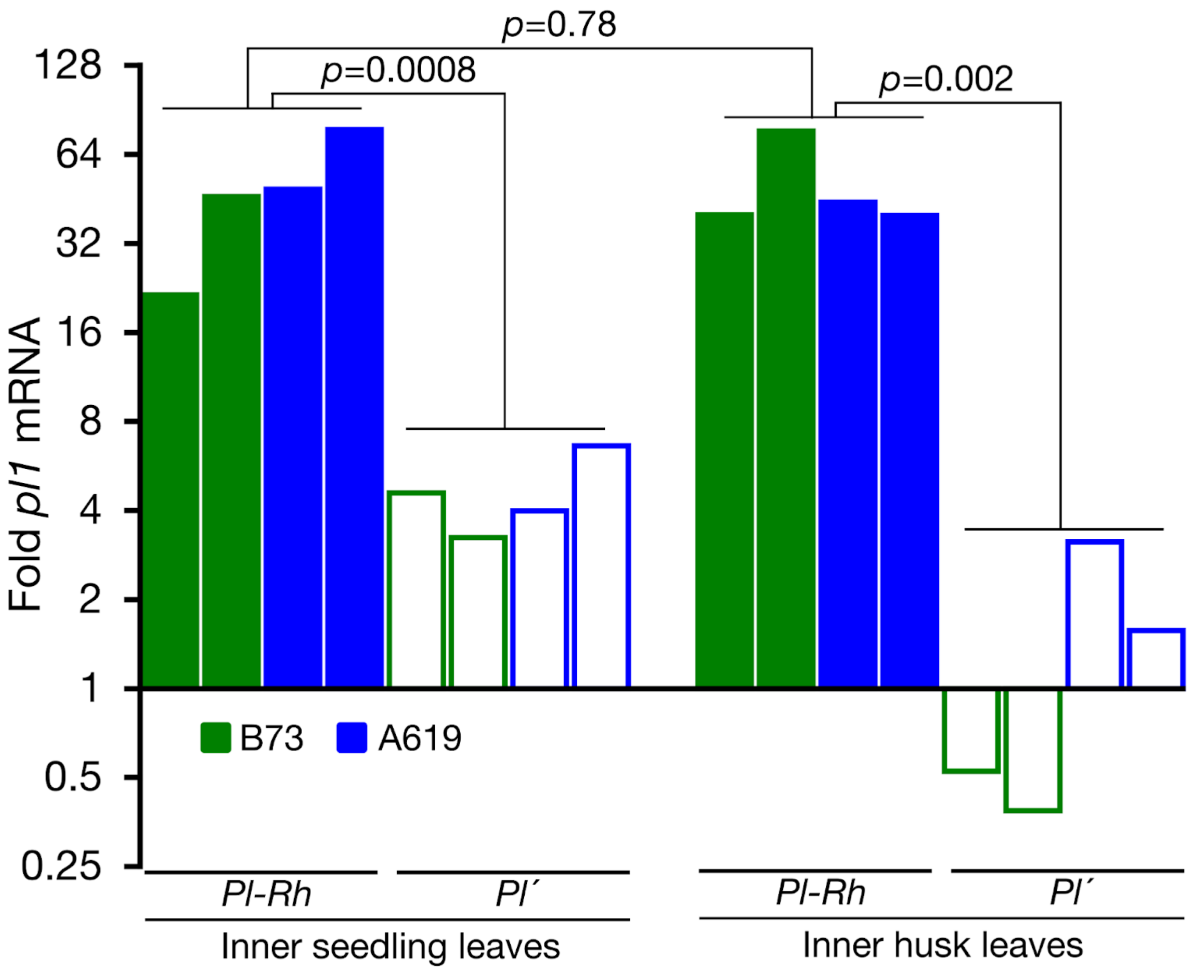

S2 Fig. *Pl1-Rhoades* mRNA expression profiles

Mean fold *pl1* mRNA ($2^{-\Delta\Delta Ct}$) in *Pl-Rh* and *Pl´* inner seedling and husk leaves relative to inner husk leaves from *Pl´* individuals measured by qRT-PCR normalized to *actin1* levels in biological duplicate plants from A619 and B73 lines.
