# Supplementary material for: Paramutation at the maize pl1 locus is associated with RdDM activity at distal tandem repeats
Source: PLoS Genet. 2024 May 30;20(5):e1011296. doi: 10.1371/journal.pgen.1011296 (PMC11166354; doi:10.1371/journal.pgen.1011296)
Supplement: S3 Fig — (A) Locations of 30 clusters called by ShortStack across the Pl1-Rhoades haplotype (blue bars) with clusters 24 and 25 which overlap the penta-repeat (B) highlighted. Arrows represent DNA transposons (light gray), Helitrons (black), and LTR retrotransposons (dark gray). (PDF) [file pgen.1011296.s003.pdf]

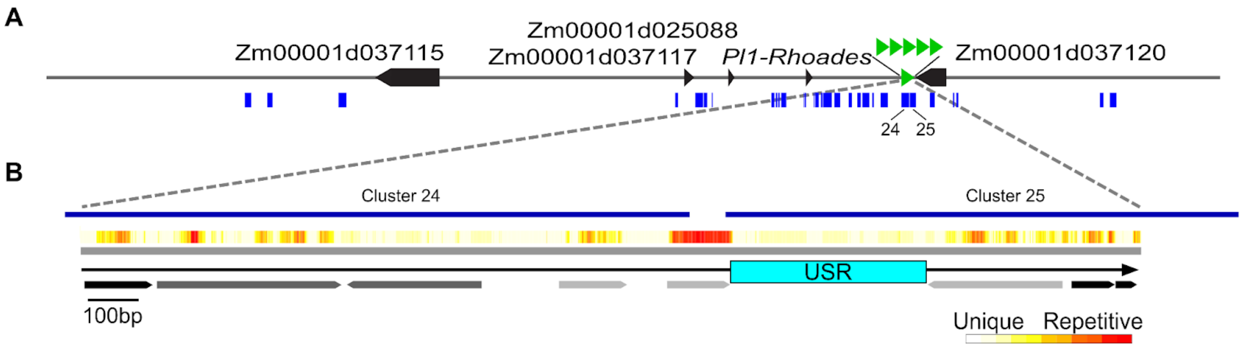

S3 Fig. sRNA clusters across the *Pl1-Rhoades* haplotype.

(A) Locations of 30 clusters called by ShortStack across the *Pl1-Rhoades* haplotype (blue bars) with clusters 24 and 25 which overlap the penta-repeat **(B)** highlighted. Arrows represent DNA transposons (light gray), *Helitrons* (black), and LTR retrotransposons (dark gray).
