# Supplementary material for: Paramutation at the maize pl1 locus is associated with RdDM activity at distal tandem repeats
Source: PLoS Genet. 2024 May 30;20(5):e1011296. doi: 10.1371/journal.pgen.1011296 (PMC11166354; doi:10.1371/journal.pgen.1011296)
Supplement: S4 Fig — Alignments of uniquely-mapping 18-30nt reads from libraries representing single Pl-Rh (A-C), and Pl' (D-F) immature cobs across the doppia fragment upstream of the Pl1-Rhoades coding sequence and the 5' region of Pl1-Rhoades exon 1 (G) in reads per million (rpm). (H) Clusters called by ShortStack. (PDF) [file pgen.1011296.s004.pdf]

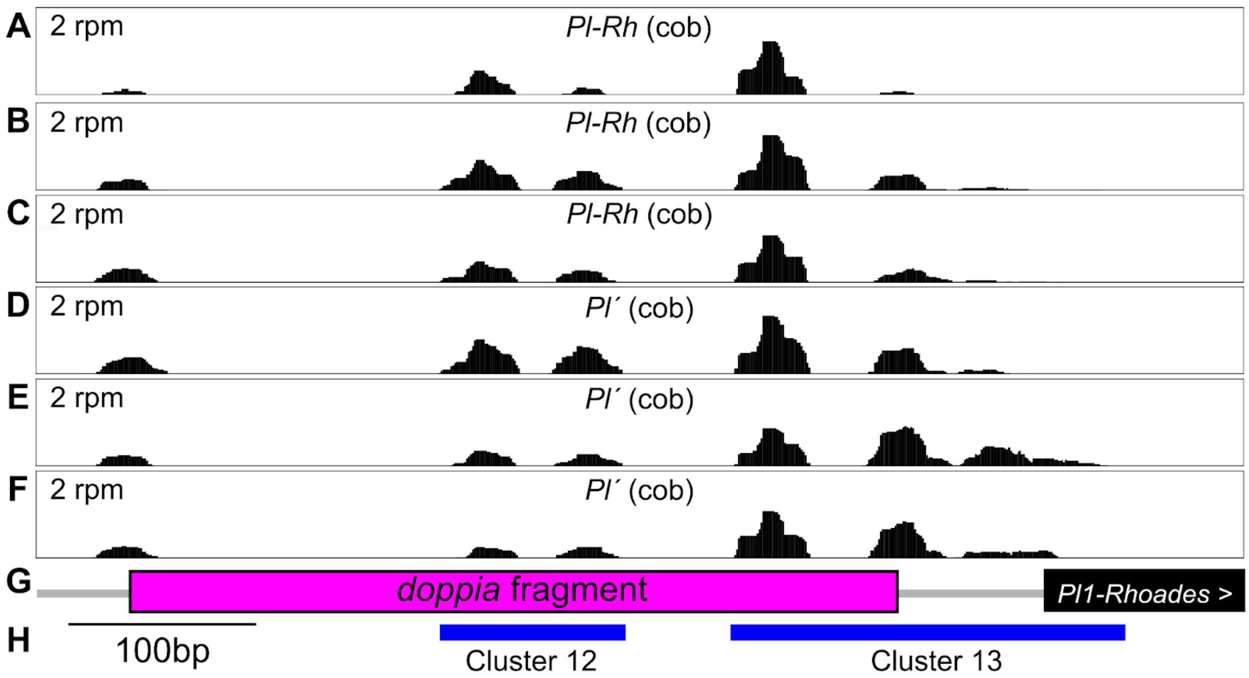

S4 Fig. Immature cob *doppia* sRNA profiles

Alignments of uniquely-mapping 18-30nt reads from libraries representing single *Pl-Rh* **(A-C)**, and *Pl´* **(D-F)** immature cobs across the *doppia* fragment upstream of the *Pl1-Rhoades* coding sequence and the 5´ region of *Pl1-Rhoades* exon 1 **(G)** in reads per million (rpm). **(H)** Clusters called by ShortStack.
