# Supplementary material for: Paramutation at the maize pl1 locus is associated with RdDM activity at distal tandem repeats
Source: PLoS Genet. 2024 May 30;20(5):e1011296. doi: 10.1371/journal.pgen.1011296 (PMC11166354; doi:10.1371/journal.pgen.1011296)
Supplement: S5 Fig — Alignments of uniquely-mapping 18-30nt reads from libraries representing single Pl-Rh (A-C), Pl' (D-F), and Pl-Rh / Pl' (G-I) immature cobs across a single repeat unit (J) in reads per million (rpm). Arrows represent DNA transposons (light gray), Helitrons (black), and LTR retrotransposons (dark gray). (PDF) [file pgen.1011296.s005.pdf]

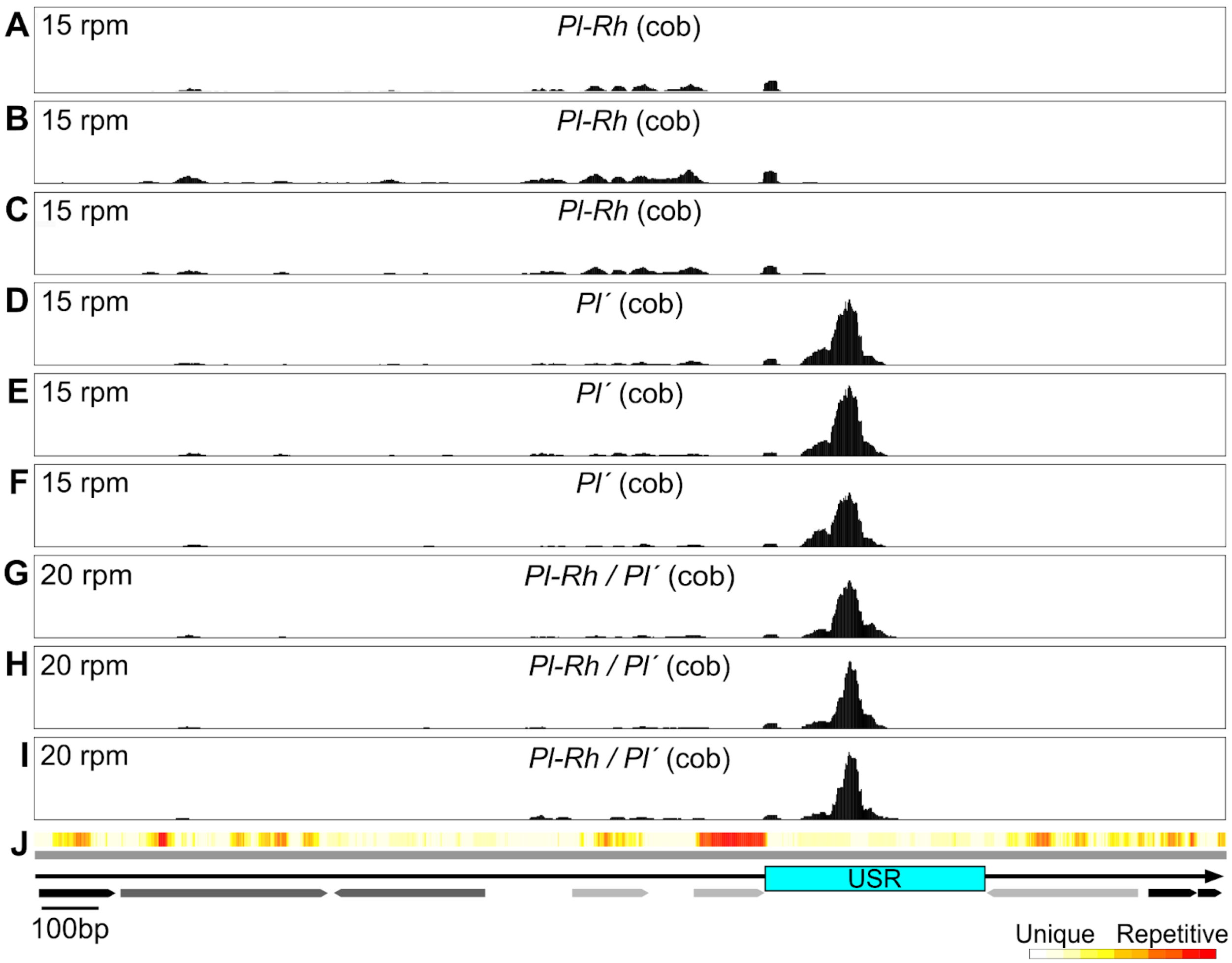

S5 Fig. Immature cob penta-repeat sRNA profiles

Alignments of uniquely-mapping 18-30nt reads from libraries representing single *Pl-Rh* **(A-C)**, *Pl´* **(D-F)**, and *Pl-Rh / Pl´* **(G-I)** immature cobs across a single repeat unit **(J)** in reads per million (rpm). Arrows represent DNA transposons (light gray), *Helitrons* (black), and LTR retrotransposons (dark gray).
