# Supplementary material for: Paramutation at the maize pl1 locus is associated with RdDM activity at distal tandem repeats
Source: PLoS Genet. 2024 May 30;20(5):e1011296. doi: 10.1371/journal.pgen.1011296 (PMC11166354; doi:10.1371/journal.pgen.1011296)
Supplement: S7 Fig — Alignments of uniquely-mapping 18-30nt reads from libraries representing single B73 inbred (A-B) immature cobs across B73 sequence (C) equivalent to the Pl1-Rhoades penta-repeat repeat unit (D) in reads per million (rpm) with structural differences highlighted (dotted lines). Arrows represent DNA transposons (light gray), Helitrons (black), and LTR retrotransposons (dark gray). Only transposons unique to B73 are labeled in (C). (PDF) [file pgen.1011296.s007.pdf]

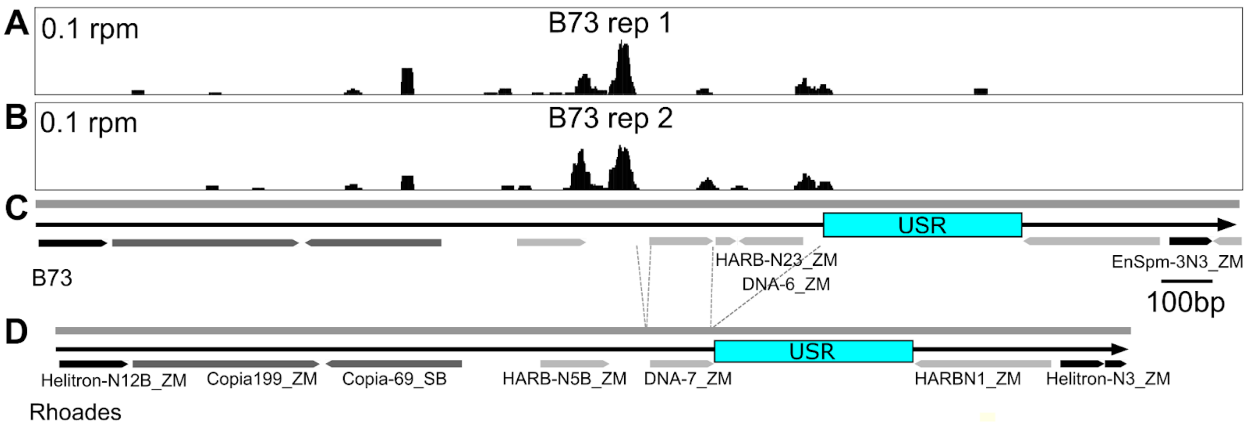

S7 Fig. B73 sRNA profiles across penta-repeat region

Alignments of uniquely-mapping 18-30nt reads from libraries representing single B73 inbred **(A-B)** immature cobs across B73 sequence **(C)** equivalent to the *Pl1-Rhoades* penta-repeat repeat unit **(D)** in reads per million (rpm) with structural differences highlighted (dotted lines). Arrows represent DNA transposons (light gray), *Helitrons* (black), and LTR retrotransposons (dark gray). Only transposons unique to B73 are labeled in **(C)**.
