# Supplementary material for: Paramutation at the maize pl1 locus is associated with RdDM activity at distal tandem repeats
Source: PLoS Genet. 2024 May 30;20(5):e1011296. doi: 10.1371/journal.pgen.1011296 (PMC11166354; doi:10.1371/journal.pgen.1011296)
Supplement: S8 Fig — Alignments of all unique and multimapping 18-30nt reads from libraries representing single Pl-Rh, Pl', and Pl-Rh / Pl' cob (A-C) and seedlings (D-F) across a single repeat unit (G) in reads per million (rpm). Arrows represent DNA transposons (light gray), Helitrons (black), and LTR retrotransposons (dark gray). (PDF) [file pgen.1011296.s008.pdf]

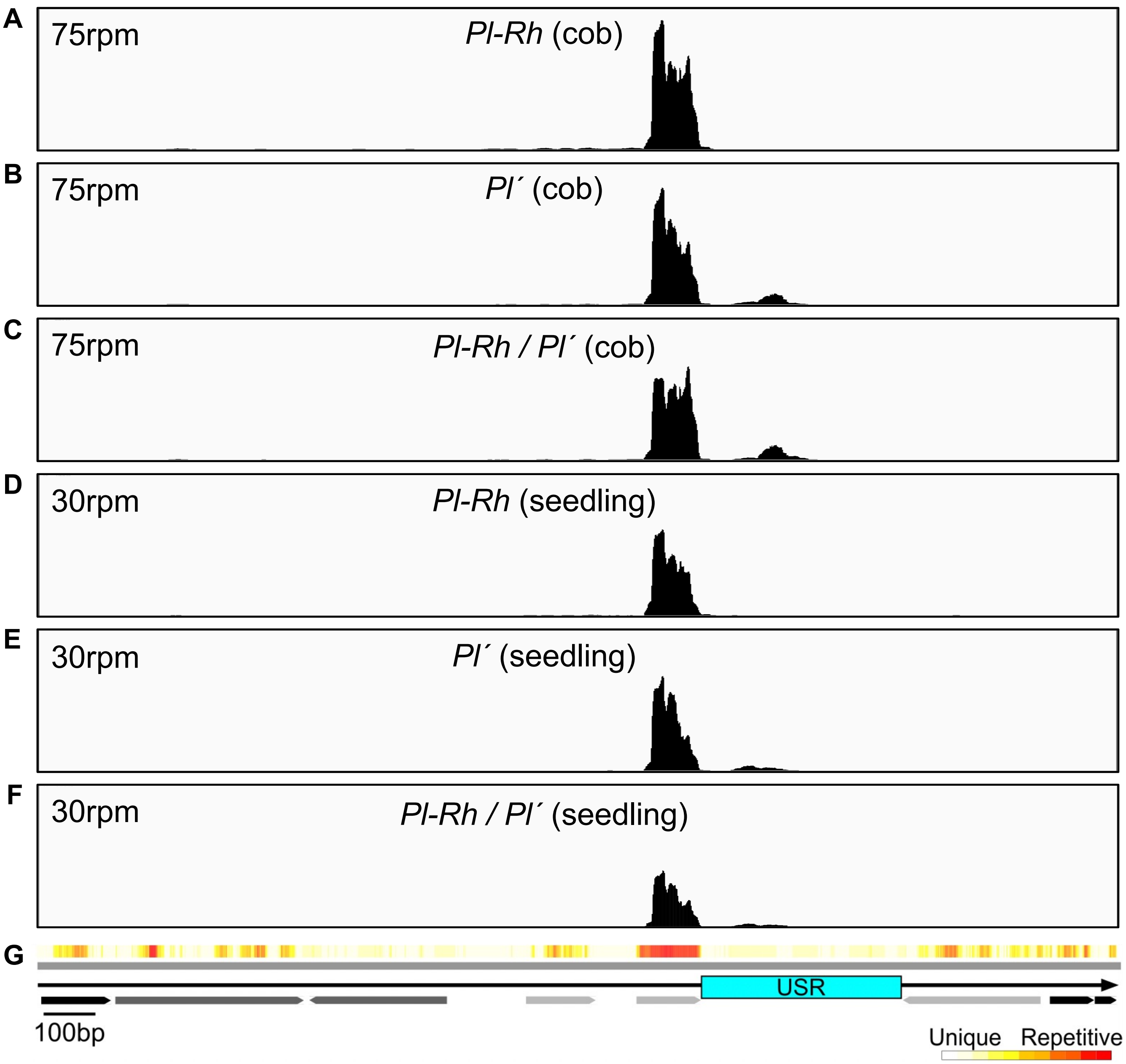

S8 Fig. Multimapping penta-repeat sRNA profiles

Alignments of all unique and multimapping 18-30nt reads from libraries representing single *Pl-Rh*, *Pl´*, and *Pl-Rh / Pl´* cob **(A-C)** and seedlings **(D-F)** across a single repeat unit **(G)** in reads per million (rpm). Arrows represent DNA transposons (light gray), *Helitrons* (black), and LTR retrotransposons (dark gray).
