# Supplementary material for: Paramutation at the maize pl1 locus is associated with RdDM activity at distal tandem repeats
Source: PLoS Genet. 2024 May 30;20(5):e1011296. doi: 10.1371/journal.pgen.1011296 (PMC11166354; doi:10.1371/journal.pgen.1011296)
Supplement: S1 Table — (DOCX) [file pgen.1011296.s009.docx]

| S1 Table. BAC sequencing statistics | | | | | | | | | | | | | | |
| --- | --- | --- | --- | --- | --- | --- | --- | --- | --- | --- | --- | --- | --- | --- |
| BAC ID | Total Reads | Polymerase Length | Polymerase Quality | Polymerase Mbp | Reads of Insert Length | Reads of Insert Quality | Reads of Insert Mbp | % ecoli reads | Maize molecules | Total molecules | Polished Contigs | Max Contig Length | N50 Contig Length | Sum of Contig Lengths |
| 60_P21 | 70817 | 6491 | 0.81 | 459.7 | 5522 | 0.82 | 391 | 12.44 | 143129 | 163482 | 10 | 136,307 | 136,307 | 252,849 |
| 25_M18 | 72163 | 6637 | 0.81 | 478.9 | 5798 | 0.82 | 418.4 | 11.4 | 144836 | 163482 | 1 | 127,734 | 127,734 | 127,734 |
| 51_M22 | 80977 | 6233 | 0.81 | 504.7 | 5223 | 0.82 | 422.9 | 14.72 | 139718 | 163482 | 3 | 107,017 | 107,017 | 110,151 |
| 53_O24 | 60206 | 6377 | 0.8 | 383.9 | 5747 | 0.81 | 346 | 8.45 | 149654 | 163482 | 1 | 139,263 | 139,263 | 139,263 |
| 161_K19 | 80358 | 5899 | 0.82 | 474 | 5045 | 0.82 | 405.4 | 16.37 | 136715 | 163482 | 3 | 131,466 | 131,466 | 134,027 |
| 192_C12 | 65913 | 5860 | 0.81 | 386.2 | 5342 | 0.81 | 352.1 | 8.65 | 149335 | 163482 | 1 | 199,360 | 199,360 | 199,360 |
