# Supplementary material for: Paramutation at the maize pl1 locus is associated with RdDM activity at distal tandem repeats
Source: PLoS Genet. 2024 May 30;20(5):e1011296. doi: 10.1371/journal.pgen.1011296 (PMC11166354; doi:10.1371/journal.pgen.1011296)
Supplement: S2 Table — (DOCX) [file pgen.1011296.s010.docx]

S2 Table. Paramutagenic properties of *pl1* haplotypes from NAM founder lines

|  | | | | | | | |
| --- | --- | --- | --- | --- | --- | --- | --- |
| (*pl1-founder* *Wx* / *T Pl´ wx*) X (*T Pl-Rh wx* / *T Pl-Rh wx*) | | | | | | | |
|  | | | | | | | |
|  | | | | | | | |
|  |  | No. of individual progeny with specific anther color scores from the respective kernel types^a^ | | | | | |
|  |  |  | | | | | |
|  |  | Glassy ( *Wx* / *wx* / *wx* )  (likely *pl1-founder* / *Pl-Rh*) | | | Opaque ( *wx* / *wx* / *wx* )  (likely *Pl´* / *Pl-Rh)* | | |
|  |  |  | | |  | | |
| Founder | Progeny ID | 1 – 4 | 5 or 6 | 7 | 1 – 4 | 5 or 6 | 7 |
|  |  |  |  |  |  |  |  |
|  | | | | | | | |
| CML52^b^ | 72376 | 6 | 4 | 7 | 30 | 0 | 0 |
| CML69 | 72393 | 2 (2) | 0 | 14 | 23 | 1 | 1 (1) |
| CML247 | 72327 | 0 | 2 | 10 | 24 (1) | 1 (1) | 3 (3) |
| CML277 | 72333 | 2 (1) | 1 (1) | 17 | 27 | 0 | 0 |
| Mo18W | 72322 | 0 | 0 | 16 | 27 | 0 | 1 (1) |
| NC350 | 182893^c^ | 0 | 0 | 14 | 7 | 0 | 0 |
| NC350 | 200722^d^ | 1 (1) | 0 | 14 | 10 | 0 | 0 |
| NC358 | 72358 | 3 (3) | 1 | 13 | 25 (1) | 0 | 0 |
| Tx303 | 72316 | 2 (2) | 1 (1) | 15 | 18 | 3 | 7 (7) |
| Tzi8 | 72303 | 6 (6) | 1 (1) | 11 | 27 | 0 | 1 (1) |
|  | | | | | | | |
|  | | | | | | | |
| ^a^ See S1 Methods for description of paramutagenicity tests. Anther pigmentation is visually assessed with a graded 1–7 scale on which values of 1–4 represent variegated *Pl´* types, 7 is full coloration typifying *Pl-Rh*, and 5 or 6 are intermediate [1]. Parentheses indicate number of progeny plants having pollen fertility phenotypes in contrast to those predicted by the assessed *wx1* genotypes. USR sequences are only found duplicated in the NC350 (2) and CML52 (3) lines.  ^b^ Previously published data [2].  ^c^ BC_1_ *Pl´* / *pl1-NC350* female and A632 *T Pl1-Rhoades* male  ^d^ BC_3_ *Pl´* / *pl1-NC350* male and B73 *T Pl1-Rhoades* female | | | | | | | |
|  | | | | | | | |

**Reference**

1. Hollick JB, Patterson GI, Coe EH, Cone KC, Chandler VL. Allelic interactions heritably alter the activity of a metastable maize *pl* allele. Genetics. 1995;141: 709–719. doi:10.1093/genetics/141.2.709.
2. Erhard KF, Parkinson SE, Gross SM, Barbour J-ER, Lim JP, Hollick JB. Maize RNA polymerase IV defines trans-generational epigenetic variation. Plant Cell. 2013;25: 808–819. doi:10.1105/tpc.112.107680.
