# Supplementary material for: Paramutation at the maize pl1 locus is associated with RdDM activity at distal tandem repeats
Source: PLoS Genet. 2024 May 30;20(5):e1011296. doi: 10.1371/journal.pgen.1011296 (PMC11166354; doi:10.1371/journal.pgen.1011296)
Supplement: S6 Table — (DOCX) [file pgen.1011296.s014.docx]

| S6 Table. Library statistics for sRNA datasets | | | | | | | |
| --- | --- | --- | --- | --- | --- | --- | --- |
| Library | Genotype | Tissue | Raw reads | Low quality (not 18-30nt) | Duplicates removed | rRNA/tRNA | Clean reads |
| H065s_1 | *Pl-Rh* | Seedling | 58,540,911 | 61.96% | 22.75% | 1.45% | 13.85% |
| H065s_2 | *Pl-Rh* | Seedling | 37,226,805 | 62.72% | 14.83% | 1.83% | 20.62% |
| H065s_3 | *Pl-Rh* | Seedling | 64,546,766 | 73.87% | 14.46% | 1.37% | 10.30% |
| H065s_4 | *Pl´* | Seedling | 92,263,047 | 57.59% | 21.52% | 3.36% | 17.52% |
| H065s_5 | *Pl´* | Seedling | 56,974,080 | 68.47% | 13.60% | 1.77% | 16.15% |
| H065s_6 | *Pl´* | Seedling | 181,184,457 | 55.39% | 32.32% | 1.49% | 10.80% |
| H086s_1 | *Pl-Rh* | Cob | 89,489,842 | 21.37% | 19.26% | 0.54% | 58.83% |
| H086s_2 | *Pl-Rh* | Cob | 83,910,901 | 21.14% | 18.84% | 0.39% | 59.64% |
| H086s_3 | *Pl-Rh* | Cob | 111,426,151 | 20.71% | 22.43% | 0.42% | 56.44% |
| H086s_4 | *Pl´* | Cob | 95,679,764 | 34.40% | 15.87% | 0.50% | 49.23% |
| H086s_5 | *Pl´* | Cob | 84,855,982 | 24.60% | 16.58% | 0.44% | 58.38% |
| H086s_6 | *Pl´* | Cob | 72,749,626 | 23.35% | 16.48% | 0.35% | 59.82% |
| H094s_1 | *Pl-Rh / Pl´* | Seedling | 86,494,648 | 59.09% | 31.92% | 0.66% | 8.34% |
| H094s_2 | *Pl-Rh / Pl´* | Seedling | 198,302,958 | 56.38% | 32.76% | 0.85% | 10.01% |
| H094s_4 | *Pl-Rh / Pl´* | Cob | 76,296,214 | 18.06% | 25.45% | 0.50% | 55.99% |
| H094s_5 | *Pl-Rh / Pl´* | Cob | 100,871,827 | 18.98% | 41.29% | 0.32% | 39.41% |
| H094s_6 | *Pl-Rh / Pl´* | Cob | 48,569,368 | 26.08% | 58.07% | 0.24% | 15.61% |
| H096s_1 | *Chd3a* | Seedling | 231,310,789 | 65.66% | 23.23% | 2.87% | 8.23% |
| H096s_3 | *Chd3a* | Seedling | 68,852,915 | 67.73% | 15.95% | 3.87% | 12.45% |
| H096s_4 | *chd3a-3* | Seedling | 65,380,316 | 58.74% | 16.75% | 3.27% | 21.23% |
| H096s_5 | *chd3a-3* | Seedling | 55,512,966 | 61.49% | 17.12% | 4.59% | 16.80% |
| H096s_6 | *chd3a-3* | Seedling | 95,032,547 | 66.34% | 13.73% | 3.87% | 16.06% |
| mut_rpd1-1 | *rpd1-1/rpd1-1* | Cob | 11,704,618 | 88.05% | | 0.16% | 11.78% |
| het_rpd1-1 | *Rpd1/rpd1-1* | Cob | 24,386,720 | 61.73% | | 0.10% | 38.16% |
| het_rpd1-1b | *Rpd1/rpd1-1* | Cob | 37,344,678 | 63.29% | | 0.13% | 36.50% |
| mut_rmr1-1 | *rmr1-1/rmr1-1* | Cob | 38,015,202 | 60.58% | | 0.07% | 39.22% |
| het_rmr1-1 | *Rmr1/rmr1-1* | Cob | 13,978,820 | 65.23% | | 0.10% | 34.64% |
| mut_rmr2-1 | *rmr2-1/rmr2-1* | Cob | 7,448,483 | 66.57% | | 0.26% | 33.14% |
| het_rmr2-1 | *Rmr2/rmr2-1* | Cob | 37,056,104 | 52.81% | | 0.08% | 47.04% |
| mut_dcl3-2 | *dcl3-2/dcl3-2* | Cob | 23,682,955 | 58.05% | | 0.07% | 41.86% |
| het_dcl3-2 | *Dcl3/dcl3-2* | Cob | 28,556,948 | 55.75% | | 0.10% | 44.13% |
| H001s | B73 | Cob | 178,338,321 | 3.62% | N/A | 0.42% | 95.96% |
| H002s | B73 | Cob | 185,712,870 | 7.07% | N/A | 0.51% | 92.42% |
