# Supplementary material for: Paramutation at the maize pl1 locus is associated with RdDM activity at distal tandem repeats
Source: PLoS Genet. 2024 May 30;20(5):e1011296. doi: 10.1371/journal.pgen.1011296 (PMC11166354; doi:10.1371/journal.pgen.1011296)
Supplement: S8 Table — (DOCX) [file pgen.1011296.s016.docx]

S8 Table. Primer sequences

| Primer name | Sequence |
| --- | --- |
| qPCR primers | |
| pl1 RT F1 (mRNA) | ACCCTGCTGCTAGCTAGCTG |
| pl1 RT R1 (mRNA) | CTGTTGCCGAGGAGCTTGTG |
| M358 (*actin1*) | CCTATCGTATGTGACAATGGCACT |
| M305 (*actin1*) | GCCTCATCACCTACGTAGGCA T |
| alt4_F | CAATATCACTGGTCAAATCCTTGCGA |
| alt4_R | TTGCACGACGAGCTAAAGACT |
| gapdh_F | CCTGCTTCTCATGGATGGTT |
| gapdh_R | TGGTAGCAGGAAGGGAAACA |
| P1_F (USR) | AGGGACTTAGAACAGCCAACAA |
| P1_R (USR) | TGCTCGTTTGTTGTAACCACC |
| P2_F (USR) | TGTTGCGTGACCGATGAGAA |
| P2_R (USR) | TAGGCCTCACCCTTCCCTC |
| P3_F (USR) | TCCAAGCACTAGTGGGTGATG |
| P3_R (USR) | AAACCGAGTCCGCTATTCCG |
| P4_F (USR) | GATAGCGAGTGATCCCCGATT |
| P4_R (USR) | CTAAGCCTTCCTGTAGTTTGATTGGC |
| 4C primers | |
| FP_H1_S2 | AATGATACGGCGACCACCGAACACTCTTTCCCTACACGACGCTCTTCCGATCTtttaaatacgccggcggagctcgatc |
| FP_H2_S1 | AATGATACGGCGACCACCGAACACTCTTTCCCTACACGACGCTCTTCCGATCTgattttaaatacgccggcggagctcgatc |
| RP | CAAGCAGAAGACGGCATACGAgcacctattaataattcacagcactgacg |
| 5mC primers | |
| 5mC_Pl1_F | AGAATGATGTTTGTTATATYAGGTT |
| 5mC_Pl1_R | CTCCAACATRTTCCAAAARCAACAC |
| Lambda_F | TTTTTATTTGGGGGAGAGGGAAGT |
| Lambda_R | TCTCCCCATCTCRCTTTCCACTCCA |
| Lambda_F2 | GGTGGGYTTTTTTGTGGGGTGAATATGG |
| Lambda_R2 | ACACTRTACTRACCRTACTCCACATCCAT |
| BAC end sequencing primers | |
| - - 1. T7 | - - 1. TAATACGACTCACTATAGGG |
| - - 1. pCC1 RP | - - 1. CTCGTATGTTGTGTGGAATTGTGAGC |
| BAC *Pl1-Rhoades*-specific primers | |
| Pl1(-15) | TGGACACCGAGAGAGAAAGAG |
| Pl1(229) | AAACCTGCACACACACCATC |
