# Supplementary material for: Paramutation at the maize pl1 locus is associated with RdDM activity at distal tandem repeats
Source: PLoS Genet. 2024 May 30;20(5):e1011296. doi: 10.1371/journal.pgen.1011296 (PMC11166354; doi:10.1371/journal.pgen.1011296)
Supplement: S9 Table — (DOCX) [file pgen.1011296.s017.docx]

| S9 Table. Enzymatic cytosine conversion efficiency of unmethylated lambda DNA | | | | | | | | |
| --- | --- | --- | --- | --- | --- | --- | --- | --- |
| Genotype | Length | Unconverted | | | Converted | | | Conversion efficiency |
|  |  | CpG | CHG | CHH | CpG | CHG | CHH |  |
| *Pl-Rh* / *Pl-Rh* | 171bp | 0 | 0 | 0 | 8 | 11 | 31 | 100% |
| *Pl´* / *Pl´* | 171bp | 0 | 0 | 0 | 8 | 11 | 19 | 100% |
| *Pl-Rh* / *Pl´* | 115bp | 0 | 0 | 0 | 5 | 10 | 11 | 100% |
