# Supplementary material for: Paramutation at the maize pl1 locus is associated with RdDM activity at distal tandem repeats
Source: PLoS Genet. 2024 May 30;20(5):e1011296. doi: 10.1371/journal.pgen.1011296 (PMC11166354; doi:10.1371/journal.pgen.1011296)
Supplement: S1 Methods — (PDF) [file pgen.1011296.s018.pdf]

Supplemental Methods

Paramutagenicity tests

The NC350 *pl1* haplotype was first made heterozygous with a *T6-9* interchange pair in which the *6L Pl´* is linked (~5cM) with a recessive *waxy1* mutation on *9L* (B73 *T Pl1-Rhoades*) and then backcrossed to the recurrent B73 female parent. Testcrosses of BC_1 and BC_3 individuals were then made using a line homozygous for the same interchange pair but carrying *Pl-Rh* (A632 *T Pl1-Rhoades* for the BC_1 or B73 *T Pl1-Rhoades* for the BC_3). Testcross progeny kernels were then sorted according to endosperm opacity to enrich for either *Pl´* / *Pl-Rh* or *pl1-NC350* / *Pl-Rh* types. Opaque endosperm types should be homozygous for the *waxy1* mutation and, by linkage, should also be homozygous for the *T6-9* interchange while glassy endosperm types are heterozygous for the *waxy1* mutation. Plants from opaque kernels (likely *Pl´ / Pl-Rh*) all displayed *Pl´*-type anthers while plants from glassy kernels (likely *pl1-NC350 / Pl-Rh*) all had *Pl-Rh*-like anthers, with one exception (see S2 Table). All freshly shed pollen grains from this exceptional individual appeared normal, which is unexpected from a plant heterozygous for a *T6-9* interchange pair that always display ~50% pollen abortion. Because this individual must therefore be homozygous for the *T6-9* interchange, the most likely explanation is that the dominant *Waxy1-NC350* allele recombined onto *T6-9* in the initial heterozygote. These data show the NC350 *pl1* haplotype did not attain any detectable paramutagenic activity, nor did it facilitate reversions of *Pl´* to *Pl-Rh* in the F_1 *pl1-NC350 / Pl´* heterozygote. Seven other NAM founder line *pl1* haplotypes having one repeat sequence were also evaluated by a similar pedigree analysis in which the *pl1* / *Pl´* F_1 plants were all testcrossed by A619 *T Pl1-Rhoades* (*Pl-Rh*). All these *pl1* haplotypes were found to be non-paramutagenic with one possible exception where 1 of 20 testcrossed glassy kernels (presumably *pl1-CML277 / Pl-Rh*) generated a plant having *Pl´*-type anthers and semi-sterile pollen (see S2 Table). This single exception may represent a rare recombinant that placed *Pl´* on the normal chromosome *6* of CML277.
