# Supplementary material for: Paramutation at the maize pl1 locus is associated with RdDM activity at distal tandem repeats
Source: PLoS Genet. 2024 May 30;20(5):e1011296. doi: 10.1371/journal.pgen.1011296 (PMC11166354; doi:10.1371/journal.pgen.1011296)
Supplement: Attachment — Submitted filename: 240408_Reviewer_Responses.docx [file pgen.1011296.s024.docx]

As you will see from the detailed comments of the three reviewers, they all value the careful and meaningful work by which you document important aspects of the paramutation at the pl1 locus, provide a lot of data on the components involved and compare it with similar cases in plants and animals. I agree with the reviewers that, though some aspects resemble the situation at other paramutation loci in maize, the differences are interesting and deserve to be pointed out. In addition to some minor suggestions for improved figures or legends, there are some points that need a bit more attention. I am aware that some of the suggestions, though reasonable, would require substantial experimental extensions, and I do not see them as essential for the publication. Other main points are easier to address, by extending the data analysis, minor additional work, or good discussions of the points. I therefore suggest that you go through the comments and provide the requested information, extend the Discussion, or answer directly to the reviewers’ comments.

We appreciate all the thoughtful contributions and have responded by providing the information we can including additional data analysis, extending the Discussion, making minor changes to the text to improve clarity, and including two additional relevant references that became available during the review process (Hövel et al., 2024 Plant Physiology and Sidorenko et al., 2024 Genetics).

 Reviewer's Responses to Questions

**Comments to the Authors:
 Please note here if the review is uploaded as an attachment.**

**Reviewer #1**: This manuscript focuses on identification and characterization of sequences required for pl1 paramutation. This is a large manuscript, with multiple sets of genetic and molecular data, that summarizes efforts of many researchers.
 Briefly, authors identified a sequence required for pl1 paramutation. This sequence is located downstream of the pl1 coding sequence and exists as an array of three to five repeats in alleles that participate in paramutation. Alleles that do not participate in paramutation have one or two repeats. Each repeat is ~2 kb in length and there is a smaller (389 bp) unique sequence (USR) embedded in each ~2kb repeat. USR exhibits following molecular characteristics; it produces polyA transcripts, accumulates low levels of 24 nt sRNAs, and exhibits differential DNA methylation. Based on 4C data, which shows an association of the penta-repeat array with the 5’ of the pl1 gene, authors propose that penta-repeats function as a pl1 enhancer. Manuscript also details analyses of sRNA accumulation in multiple mutant backgrounds and authors extensively discuss the role of RdDM in pl1 paramutation. While there are some pl1 paramutation-specific molecular characteristics, majority of findings are consistent with those previously reported for paramutation at r1, b1 and p1 loci.
 The similarities to other cases of paramutation in maize in no way negate the value of this manuscript. Uncovering and documenting similar trends between different cases of paramutation is essential for forming a comprehensive understanding of how paramutation is regulated. Collectively, this and prior studies demonstrated that repeated enhancer arrays carrying unique locus-specific DNA are the key feature of the phenomenon of paramutation. Based on this, I think that the data presented in this paper are of interest to a broad circle of researchers working in the area of epigenetics. I recommend this manuscript for publication, after minor issues detailed below are addressed.

Minor comments:
 Page 24, line 428, says “paramutagenic pl1 haplotypes…”. Should it be “paramutagenic p1 haplotypes”? The references that follow this phrase are for p1, not pl1 locus.

Thank you for the careful reading! We have deleted the “l”.

Page 27, Lines 487-497. It would help if it was made clearer how this paragraph relates to the experimental data in the manuscript. It does not seem to add much to the discussion.

The paragraph comes after previous consideration of chromosome-based changes that might be responsible for the meiotic inheritance of paramutagenicity so we consider it important to discuss the potential for sRNA-based inheritance – especially as there is evidence to support sRNAs as paramutagenicity vectors in metazoans. We have added the following topic sentence to that paragraph to better connect these two views.

“Gametic sRNAs may also contribute to the inheritance of repressed *Pl´*.” (Line 533)

Figure 2. Two suggestions here:1) What “Total” in panel A is showing? Because of the Y axis scale, it does not look as it shows the sum of panels B and C. Is it the average of panels B and C? Please, clarify in the figure legend.

We have changed “Total” to “Combined” as a more accurate description. Neither sum nor average is accurate because the reads are added together but then normalized to the reads per million of the combined datasets.

2) I suggest adding tracer lines to connect the expanded diagram in panel E to the corresponding section of DNA in panel D. This would help with faster comprehension of the figure.

This is a good suggestion and we have modified the figure accordingly.

Figure 8. I propose adding tracer lines to connect diagram of methylation sites in panels A-C to the location of the sequence assayed in panel D (bracketed black line below the USR box).

We agree and have modified the figure accordingly.

S. Fig 3. I propose to add tracer lines that would indicate which region of sequence in panel A is expanded in panel B. This modification would make it easier to understand the figure.

We agree and have modified the figure accordingly.

**Reviewer #2**: Deans et al., report on the characterization of a distal cis-regulatory element involved in paramutation at the pl1 locus that is located approximately 14 kb downstream of the pl1 polyadenilation site and is formed by five copies of a repetitive fragment organized as a tandem repeat. The authors present a thorough and comprehensive review of the current knowledge on paramutation in plants that is mechanistically contrasted in the discussion with information coming from animal systems (this was a pleasant addition). There is a lot of work included in this manuscript. The authors performed chromatin conformation analysis indicating that the penta repeat comes in close proximity to the promoter of pl1. Analysis of sRNA-seq data in Pl-Rh an Pl’ show differential accumulation of sRNAs mapping to the penta repeat in two different tissues (cob and seedlings). Analysis of the expression pattern of sRNAs mapping to the penta repeat in mutant backgrounds of the RNA-directed DNA methylation pathway (RdDM) genes indicate a role for classical RdDM components including small RNAs (sRNAs), NRPD1 (POL4), DICER-LIKE 3 (DCL3 ) and other non-classical such as CHROMODOMAIN HELICASE DNA-BINDING 3 (CHD3). Taken together, the authors present a model (based on the model proposed for paramutation at the b1 locus).


 The data is interesting since the genetic requirements for paramutation at pl1 are different relative to paramutation at the b1 locus, including the molecular nature of the tandem repeats and the function of genes that are unique to pl1 for example CHD3 is not required for paramutation at the b1 locus. The authors propose that paramutation at the pl1 locus involves a mechanism based on RdDM or an RdDM-like mechanism that invokes transcription by RNA POL II in Pl-Rh and transcription by RNA POL IV in Pl’. Does the observation that in Arabidopsis POLIV-dependent transcripts lack a 3’-poly(A) tail has been shown to be the same in maize?.

The reviewer is correct that it remains unknown whether maize RNAP IV or RNAP V transcripts lack 3´-poly(A) tails and we have now added this caveat in the discussion section by 1) replacing RNAP II with “polyadenylated” in the following passage (lines 417-420):

“whereas the abundance of both penta-repeat polyadenylated transcripts and RNAP IV-dependent sRNAs distinguish *Pl-Rh* from *Pl´*.”

and 2) adding the following paragraph (also in response to Reviewer 3’s interest) in the discussion:

“The polyadenylated USR-containing RNAs associated with the active *Pl-Rh* state indicate this region is transcribed but the origin(s), direction(s), and length(s) of these RNAs remains unknown. Because neither Arabidopsis RNAP IV nor RNAP V produce polyadenylated RNAs (Blevins et al., 2015; Li et al., 2015; Zhai et al., 2015; Wierzbicki et al., 2008; Wendte et al., 2017), we infer that these USR-containing transcripts are RNAP II-derived. Whether this transcription reflects a more open chromatin nucleosome-free configuration region, however, remains equivocal. Because the B73 AGPv4 annotated PAS of the adjacent gene immediately downstream of the penta-repeat is less than 100bp from the end of the last repeat, one idea is that pre-termination transcription continues into the repeat array and a downstream PAS is used. Alternatively, the penta-repeat RNAs could initiate within USR sequences or from its resident TE fragments. In either case, a nascent RNA containing USR sequences and/or the adjacent highly repetitive DNA-7_ZM TE could serve as an essential scaffold transcript for the initial recruitment of RNAP V via sRNA-directed argonaute complexes (Sigman et al., 2021). Hence, a paramutable *Pl-Rh* state may require such polyadenylated penta-repeat RNAs for paramutation initiation while a paramutagenic *Pl´* state likely uses RNAP V-sourced transcripts to maintain sRNA biogenesis.” (Lines: 425-441)

In Arabidopsis POLIV-dependent transcripts are produced from both strands. Did you test this at the USR in Pl-Rh and Pl’?

Although the Figure 6H-6M data make it clear that both sense and antisense 23/24nt products represent both strands, we cannot state that the primary RNAP IV transcripts are produced from bidirectional transcription. We did point out that the observed cob 23mer bias for the bottom strand supports a preferred direction of RNAP IV transcription (Lines 336-337).

Given there is no experimental evidence supporting a role for CHD3 in positioning RNA POLIV, What prevents POL4 from transcribing in Pl-Rh?.

24mer production from the adjacent 3’ TE in chd3a mutants is solid genetic evidence for CHD3a acting directly or indirectly to prohibit RNAP IV action at those TE sequences. We have added the word “action” on line 274- 276

“these findings imply that CHD3a action normally positions RNAP IV at the USR in a paramutagenic *Pl´* state.”

- to clarify that this observed effect on RNAP IV does not infer a direct protein-protein interaction. A role for CHD3a in directing where RNAP IV functions at a genome wide level has also been shown (Deans et al., 2020 PLoS Genetics).

Our hypothesis for why RNAP IV is not transcribing USR sequences in *Pl-Rh* is that transcription of the USR by RNAP II is precluding RNAP IV recruitment – this idea is explicitly stated in the first paragraph of the discussion. A similar polymerase “competition” hypothesis has beenproposed since 2009 (Hale et al., 2009 PLoS Genetics). Additionally, because the USR in *Pl-Rh* appears unmethylated it is not likely that RNAP IV would be recruited to this sequence.

CHD3 is not required for paramutation at the b1 locus so what is special about the penta repeat in pl1 that requires non-general components such as CHD3 that might be interacting with RNA POLIV?

This is an interesting question but unaddressable with the current set of experiments. The *b1* hepta-repeats have no TE fragments but whether CHD3a is specifically acting on TE sequences and therefore not affecting the *B1-I* enhancer is mostly speculative.

We have added the following paragraph to the Discussion to bring up this interesting question for future investigation:

“The distinct sequences and structural arrangements of the *B1-I* and *Pl1-Rh* distal repeats may be related to the observed differences in allele stabilities and locus-specific RMR requirements. Neither RMR1 nor RMR12 repress *B´* (Hale et al., 2007; Deans et al., 2020). Each individual unit of the *b1* hepta-repeat (853bp) consists entirely of unique sequence (Stam et al., 2002), while the 2092bp penta-repeat unit is mainly consisting of TE fragments with an embedded 390bp unique sequence. Unlike *B´*, where heritable reversions to a fully expressed *B-I* state have not been observed (Coe 1966; Patterson et al., 1993; Chandler et al., 2000), the *Pl´* state is less stable and can revert to *Pl-Rh* when either hemizygous (Hollick and Chandler, 1998), heterozygous with certain recessive alleles (Hollick and Chandler, 1998; Gross and Hollick, 2007) or in the persistent absence of RMR1 (Hollick and Chandler 2001; Barbour et al., 2012), RPD1 (Hollick et al., 2005), CHD3a (Deans et al., 2020), or DCL3 (Narain et al., 2023). Whether the size, copy number, location, and/or composition of these *b1* and *pl1* repeats contribute to the observed differences in allele behaviors and mechanistic requirements remains to be explored.” (Lines 559-573)

Does the POLIV isoform that is proposed to interact with CHD3 also includes MOP2/RMR7 or a different second largest subunit?

To be clear, we made no claim that CHD3a directly interacts with RNAP IV.

This too is an interesting question but unaddressable with the current set of experiments. We stated on lines 358-359: “We therefore conclude that the majority of USR sRNAs depend on RNAP IV, though the specific RNAP IV subtype(s) acting there remains unknown.”

Future USR sRNA profiling of *rmr7* mutant cobs and seedlings will be a great set of experiments to ask whether RNAP IVa and/or IVb acts at the USR. Given that anther and plant pigmentation due to *Pl´* is enhanced to *Pl-Rh*-like levels in *rmr7* null mutants (Stonaker et al., 2009 PLoS Genetics), we do expect RNAP IVa to be USR engaged in those tissues.

The accumulation pattern of the sRNAs mapping to the USR in the same tissue (immatue cobs) across the different mutant backgrounds makes it difficult to understand the actual role of these sRNAs. Paramutation is not affected in heterozygous Rmr1 / rmr1-1 and Rmr2 / rmr2-1 individuals. However there is a clear reduction (around 50% of maybe more) in the levels of sRNAs mapping to the USR in Rmr1 / rmr1-1 and Rmr2 / rmr2-1. How does the model account for this?

While the *Pl´* vs *Pl-Rh* sRNA and 5mC profilings used isogenic B73 materials, the mutant vs non-mutant cob sRNA comparisons reflect a variety of different genetic backgrounds. We do not expect read-depth normalized abundances across the repeat unit to be identical in all different genetic backgrounds so feel such comparisons are insufficiently quantitative to make claims about sRNA thresholds. The qualitative presence/absence patterns are nonetheless conserved for all non-mutant/mutant comparisons.

Do the differences in 5mC observed in Pl-Rh and Pl’ seedlings are the same in wild-type cobs and cobs from Rmr1 / rmr1-1 and Rmr2 / rmr2-1 heterozygous individuals?

We are currently working on a separate manuscript that extensively investigates the 5mC correlations from paramutation establishment and reversions. As recognized by the reviewer, there is already a lot of work described in this manuscript. Adding additional 5mC profiling in this manuscript becomes too much as there are many mutants, developmental timepoints, and specific breeding scenarios to assess.

What is the accumulation pattern of sRNAs mapping to the USR in cobs from Chd3a / chd3a-3 individuals?

We have not generated sRNA profiles from *chd3a* mutant cobs because developmental staging of these tissues is virtually impossible (see Deans et al., 2020 PLoS Genetics). *chd3a* mutant cobs are severely delayed and dwarfed compared to non-mutant siblings making coherent evaluations of “like” tissues dependent on staging with molecular markers – which is a project all in itself.

Since loss-of-function of ZmDCL3 results in a modest effect on global 5mC levels and sRNAs mapping to the USR are dramatically depleted in dcl3-2 / dcl3-2. What are the 5mC levels at the USR in the dcl3-2 / dcl3-2 mutant background?

We are a bit unclear about this question given that Narain et al., (2023 BioRxiV) did not evaluate global 5mC. Several specific regions showed no 5mC loss, which was unexpected, but a global survey was not conducted. Here again, this is an interesting question and we are currently working on a separate manuscript that extensively investigates the USR 5mC correlations – including in the *dcl3* mutants.

What is the accumulation pattern of sRNAs mapping to the USR and the 5mC levels at the USR in a MOP2/RMR7 mutant background?

Another interesting question, and we are currently working on a separate manuscript that extensively investigates the USR 5mC correlations – including in *rmr7* mutants.

**Reviewer #3:** In this manuscript Deans et al. convincingly identify the regulatory region for maize pl1 paramutation: distal tandem repeats likely acting as enhancers. The reported similarities and differences with other loci susceptible to paramutation in maize and other species are an important step to better understand the mechanism of this epigenetic phenomenon. The manuscript is very clear and the data well presented. I have found no major issues with the current manuscript and therefore only provide suggestions for improvements

We appreciate the supportive words.

suggestions:

chromatin conformation: Could you perform 4C for the Pl’ allele? It would be interesting to see whether the silenced form of the tandem repeats still interacts with the pl1 promoter. A reciprocal  4C from the viewpoint of the USR would also inform on the potential for the tandem repeat to affect other genes in the region, and check those genes’ expression for potential paramutation.

This is a good suggestion and one that should be pursued at a later time. Ideally, the 4C experiments should be replicated with both *Pl´* and *Pl-Rh* at the same time. Our data was generated by a previous PhD student, and we would need to dedicate at least 6 months of graduate student or postdoc time to reestablish the methodology and expertise with which to conduct and analyze these difficult assays.

sRNA profiles: it would be helpful to also present the alignment results with the multi-mapping reads, to better understand what the full population of sRNAs at this locus looks like. Possibly on a Pl1-Rh haplotype-substituted B73 reference. Can you comment on the observed 24 nt RNA levels compared to other clusters genome-wide? It doesn’t look like they are super abundant, which is compatible with the mostly symmetric mC and the fact that paramutation loci aren’t predicted by massive amounts of 24nt sRNAs (at least in the tissues studied)

This is a fair request and we have now repeated the analysis with all multimapping reads and presented the results in Supplemental Figure 8.

The results do highlight a massive sRNA peak over the DNA-7 ZM TE just 5’ of the USR but nothing else of note. We have added the following paragraph in the results section to introduce this finding:

“We next previewed the entire penta-repeat unit sRNA landscapes by including both unique and multi-mapping reads from *Pl-Rh*, *Pl´* and *Pl-Rh /* *Pl´* in cob and seedling (Methods). In all datasets, profiles were dominated by a similar multimapping read cluster precisely over the repetitive DNA-7_ZM TE, dwarfing the adjacent USR read peak (see S8 Fig). While the genomic source of these TE sRNAs is unknown, they could contribute to recruiting RNAP IV or V in the *Pl´* state.” (Lines 343-348)

We don’t have cluster calls from a substituted whole genome. However, the rpm values from each of the 30 clusters called across the 192kb BAC sequence (see S2 File) show that cluster 25 (the one overlapping the USR) has the highest abundance of any of the clusters in the *Pl´* and *Pl-Rh / Pl´* datasets from both cob and seedling (added to the results in lines: 321-323). While this is not a genome-wide assessment, it suggests that sRNAs from this cluster are fairly abundant (20-30rpm). There certainly could be other tissues/stages where sRNA production from the USR would be higher and we plan to investigate sRNA production and methylation in other tissues in a future manuscript.

I am intrigued by the USR Pol II-dependent RNA: is it some kind of long non-coding RNA? Can public RNA-seq data be mined to infer its boundaries? Can you comment on its absolute expression level? For instance if it’s basically 0 in Pl’, is a 15-fold increase in Pl-Rh still practically 0? Full-length cDNA + capture + long-read sequencing could also help resolve what’s being transcribed there.

To be fair to Reviewer 2’s comment above, we do not know if this/these polyA+ transcript(s) are RNAP II products as they could be derived from RNAP IV and/or RNAP V. Neither public mRNA-seq data, nor extensive internal unpublished mRNA-seq datasets have sufficient depth to identify any mRNA species containing USR sequences. The expression levels are relatively low, even in the *Pl-Rh* state, but they are also detectable in the *Pl´* state. Raw qRT Ct values are now provided in supplemental dataset (S5 File), however determining absolute expression from Ct values alone is speculative since primer sequences, template, and reaction conditions influence amplification efficiency.

As mentioned above in response to Reviewer 2, we have added the following paragraph in the discussion to highlight the potential involvement of this USR-containing transcript in the initiation of paramutation:

“The polyadenylated USR-containing RNAs associated with the active *Pl-Rh* state indicate this region is transcribed but the origin(s), direction(s), and length(s) of these RNAs remains unknown. Because neither Arabidopsis RNAP IV nor RNAP V produce polyadenylated RNAs (Blevins et al., 2015; Li et al., 2015; Zhai et al., 2015; Wierzbicki et al., 2008; Wendte et al., 2017), we infer that these USR-containing transcripts are RNAP II-derived. Whether this transcription reflects a more open chromatin nucleosome-free configuration region, however, remains equivocal. Because the B73 AGPv4 annotated PAS of the adjacent gene immediately downstream of the penta-repeat is less than 100bp from the end of the last repeat, one idea is that pre-termination transcription continues into the repeat array and a downstream PAS is used. Alternatively, the penta-repeat RNAs could initiate within USR sequences or from its resident TE fragments. In either case, a nascent RNA containing USR sequences and/or the adjacent highly repetitive DNA-7_ZM TE could serve as an essential scaffold transcript for the initial recruitment of RNAP V via sRNA-directed argonaute complexes (Sigman et al., 2021). Hence, a paramutable *Pl-Rh* state may require such polyadenylated penta-repeat RNAs for paramutation initiation while a paramutagenic *Pl´* state likely uses RNAP V-sourced transcripts to maintain sRNA biogenesis.” (Lines: 425-441)

DNA methylation: a higher-throughput method would be great. If there are 5 repeats, the 10 Sanger reads could all be from the same nucleus in theory! At a minimum, you could directly sequence the PCR product. If DNA  input is not limiting, I’d recommend Cas9-targeted nanopore sequencing, you could get 100X depth, encompass the whole tandem array and find interesting patterns from repeat to repeat, and you can add guide RNAs for other interesting regions like the pl1 promoter in the same experiment. The public long-read nanopore datasets might also enable DNA methylation analysis for this region in B73 at least.

This is a great suggestion and we agree that it will be essential to conduct future 5mC profiling in a more high-throughput fashion. We do not have more of the original samples used for the profiling presented in this manuscript so cannot simply reamplify and sequence the PCR products. We are aware of selective nanopore sequencing methods but there is still concern that the basecalling accuracy for 5mC – especially in CHH contexts – is still rather poor, and establishing these techniques is also time consuming.

l. 168 add “in convergent orientation” to make it clear the tandem repeat is between the two genes.

We added the word “convergent” in “3´ of the next convergent gene model” (Lines: 180-185)
